# Ergonomic Consideration in Pillow Height Determinants and Evaluation

**DOI:** 10.3390/healthcare9101333

**Published:** 2021-10-07

**Authors:** Jia-Xing Lei, Peng-Fei Yang, Ai-Ling Yang, Yan-Feng Gong, Peng Shang, Xi-Chen Yuan

**Affiliations:** 1 School of Life Sciences, Northwestern Polytechnical University, Xi’an 710072, China; ljx18235109697@mail.nwpu.edu.cn (J.-X.L.); yangpf@nwpu.edu.cn (P.-F.Y.); shangpeng@nwpu.edu.cn (P.S.); 2 Key Laboratory for Space Bioscience and Biotechnology, Institute of Special Environment Biophysics, Northwestern Polytechnical University, Xi’an 710072, China; 3 Shenzhen Zhengjing Technology Limited Liability Company, Shenzhen 518057, China; yangal@hepalink.com (A.-L.Y.); yf_gong001@163.com (Y.-F.G.); 4 Research & Development Institute in Shenzhen, Northwestern Polytechnical University, Shenzhen 518057, China; 5 Ministry of Education Key Laboratory of Micro/Nano Systems for Aerospace Northwestern Polytechnical University, Xi’an 710072, China

**Keywords:** pillow height, cervical spine alignment, body dimensions, contact pressure, muscle activity

## Abstract

(1) Background: Sleep problems have become one of the current serious public health issues. Pillow height affects the alignment of the cervical spine and is closely related to the mechanical environment of the cervical spine. An appropriate pillow height can provide adequate support for the head and neck to reduce the stress in the cervical spine and relax the muscles of the neck and shoulder, thereby relieving pain and improving sleep quality. (2) Methods: We reviewed the current trends, research methodologies, and determinants of pillow height evaluation, summarizing the evidences published since 1997. In particular, we scrutinized articles dealing with the physiological and mechanical characteristics of the head-neck-shoulder complex. (3) Results: Through the investigation and analysis of these articles, we presented several quantitative and objective determinants for pillow height evaluation, including cervical spine alignment, body dimension, contact pressure, and muscle activity. The measurement methods and selection criteria for these parameters are described in detail. However, the suggested range for achieving optimal cervical spine alignment, appropriate pressure distribution, and minimal muscle activity during sleep cannot yet be identified considering the lack of sufficient evidence. Moreover, there remain no firm conclusions about the optimal pillow height for the supine and lateral positions. (4) Conclusions: A comprehensive evaluation combining the above determinants provides a unique solution for ergonomic pillow design and proper pillow height selection, which can effectively promote the public sleep health. Therefore, it is necessary to develop a reasonable algorithm to weigh multiple determinants.

## 1. Introduction

Sleep is an indispensable part of the activities of human life and accounts for approximately one-third of the total life span. Sleep plays an important role in the improvement of immunity, memory, and recovery of physical vitality [1]. However, sleep disorders and problems are common. According to a survey conducted by the World Health Organization, the incidence of insomnia among Chinese is as high as 38% [2]. Sleep problems have become a serious public health issue, which can have negative impacts on mental and physical health [3,4]. Sleep systems and support are considered key environmental factors affecting the comfort of the body during sleep [5]. Improper sleep systems can cause concentrated pressure and musculoskeletal discomfort, which are the main causes of sleep deprivation [6,7].

The pillow is an important part of the sleep system, which is functioned to support the head and neck, further maintain the physiological curvature of the cervical spine, relax the neck muscles, reduce the pressure on the cervical intervertebral disk, and optimize the load distribution between the intervertebral disks [8,9]. The lack of support for the head and neck may adversely alter cervical spine alignment and cause musculoskeletal problems, including neck pain, scapular pain and muscle stiffness [10,11,12].

Pillow height is an important characteristic of pillows, which affects the cervical spine alignment and is closely related to the mechanical environment of the cervical spine. However, selecting an appropriate pillow height is difficult. Most studies usually evaluate pillow height based on subjective methods, such as subjective comfort evaluation and questionnaires [10,13,14,15]. However, subjective evaluation is easily affected by age, gender, individual perception, and long-term sleeping habits, which makes the conclusion disputable and unreliable. In addition to subjective evaluation, pillow height has also been shown to affect cervical spine alignment, pressure distribution in the cranial and cervical regions, as well as muscle activity of the neck and shoulder [16]. Firstly, the ideal pillow height can maintain the physiological curvature of the cervical spine by providing adequate support for the head and neck [9]. Secondly, the difference in the forces supporting the head and neck reflects the capability to maintain cervical spine alignment and has been regarded as a key factor in pillow height evaluation [17,18]. Finally, some previous studies have examined pillow height in relation to muscle activity and found that pain and discomfort are associated with higher and longer electromyographic activity [19].

Many hypotheses have been proposed regarding the optimal pillow height, but there are still no firm conclusions. Therefore, some quantitative and objective indicators need to be put forward to scientifically evaluate the appropriateness of pillow height, and pillow manufacturers also need supporting experimental data to optimize pillow design. In this review, we summarized the measurement methods and determinants used to evaluate pillow height based on the effects of the physiological and mechanical characteristics of the head-neck-shoulder complex. This review will further improve the current understanding of the potential role of pillow height on cervical spine discomfort during sleep and stimulate the formalization of objective standards for pillow height evaluation.

## 2. Methods

### 2.1. Search Strategy

An article search was conducted using a combination of the following keywords: “pillow height”, ”cervical spine alignment”, “body dimensions”, “contact pressure” and “muscle activity”. Several databases were accessed, including PubMed, Web of Science, ScienceDirect and Scopus. Studies conducted from 1997 to 2021 and published in peer-reviewed journals in English were considered. Meanwhile, the study field is limited to “Life science”, “Biomedicine”, and “Health professions”.

### 2.2. Study Selection

**Figure 1:**
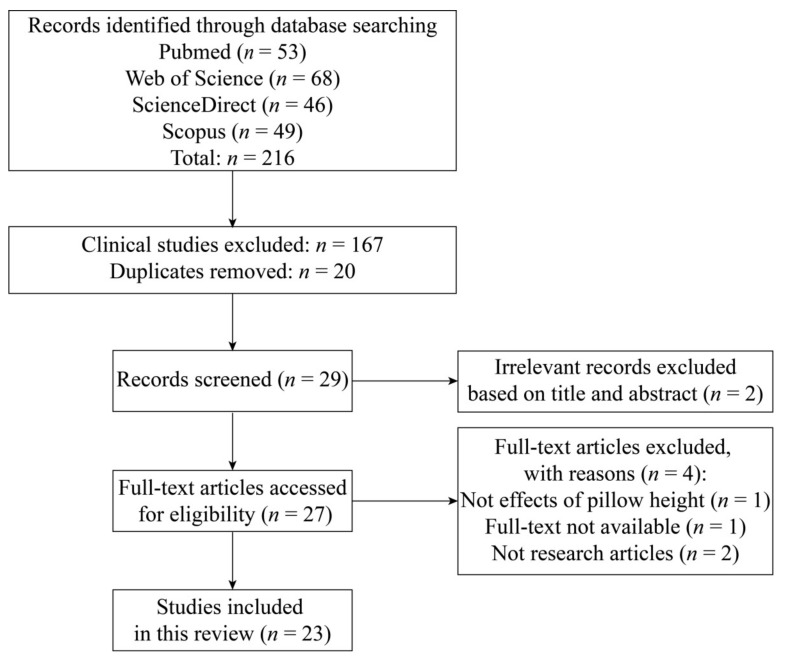
PRISMA flow chart of study selection in this review.

The study selection included primary screening based on title and abstract by two independent reviewers and a second screening based on full text. When any discrepancies occurred in the results of the search processes, two more reviewers intervened, and the final outcome was settled by discussion. Clinical articles were excluded, such as laryngoscopy, anesthesia and tracheal tube intubation. Articles unrelated to pillow height were excluded. Figure 1 shows the PRISMA flowchart.

### 2.3. Data Extraction

The details of some important articles were included in this review. The following data were extracted from each study: (1) Table 1: Selection of subjects and pillow samples; (2) Table 2: Measurement methods and outcomes; (3) Table 3: Study scope and key findings. 

### 2.4. Data Synthesis

The articles reviewed can be divided into the following four categories: (1) the effects of pillow height on cervical spine alignment; (2) the relationship between pillow height design and body dimensions; (3) the effects of pillow height on the pressure distribution in the cranial and cervical regions; (4) the effects of pillow height on the electromyographic activity of the neck and shoulder muscles.

## 3. Results

### 3.1. Subjects and Population

All the participants were healthy. Exclusion criteria included sleep disorder, spinal injury, and musculoskeletal disorders related to the spine, such as neck pain, lumbar disease, spinal symptoms, or their treatments. Although the study by Chen and Cai [11] did not clearly point out the exclusion criteria for subjects, the subjects were young students, therefore, we considered them to be healthy. No study indicated how subjects should be evaluated to be free of these symptoms, so we default on a hospital diagnosis. However, it is difficult to diagnose sleep disorders and musculoskeletal discomfort. Therefore, the current study was not sufficiently rigorous in the selection of subjects. In addition, Cai and Chen [21] also excluded participants who had important work to do the next day, so that they could avoid sleeping with stress. As quinquagenarian women were studied, Li et al. [23] excluded those who could not complete the questionnaire independently.

Three studies did not indicate the gender of subjects, six studies investigated males and females, and one study investigated only females. For ease of comparison, four studies had equal numbers of men and women. It was found that pillow heights are different for men and women because of the build of the body [11,21]. Therefore, male and female volunteers should be recruited separately and included in the analysis to avoid the one-sidedness of a single sex.

Two studies did not indicate the age of the subjects, while the rest were adults. The subjects in the six studies were all young people, most of whom were 20–30 years old. As most of the studies reviewed were conducted in universities, the subjects were mainly college students. Only one study involved quinquagenarian women, with a mean age of 53.74 ± 6.80 years [23]. None of the studies compared the outcomes of young and elderly people. In fact, due to differences in spinal curvature, joint stiffness, and subjective perception between the young and elderly, the pillow height suitable for them may vary.

Body shape may affect the comfort perception of the subjects and the body–pillow contact pressure at the same pillow height. Because the purpose of the research related to body dimension is to design pillows suitable for sleeping according to different body shapes, the differences in body shape need not be considered. In a study on constructing an ideal pressure distribution pillow model for head and neck support, Li et al. [2] selected participants with similar physical fitness levels to avoid the effect of different body shapes on the outcomes. This is of great significance to the accuracy and validity of the experimental results. In addition, the remaining reviewed studies did not strictly limit the body shape of the subjects.

### 3.2. Pillow Samples

Among all articles, three described the pillow samples vaguely, and the remaining seven studies focused on pillow materials and pillow shapes. With regard to the pillow material, foam is considered to be the best material for supporting the cervical spine, which can relieve waking pain and improve sleep quality [25]. At present, foam pillows are a common product in the market and are among the most popular pillows among pillow users [26]. Five studies used foam as a filling material for pillows, such as urethane foam, foam rubber, and polyurethane foam. Foam is soft and comfortable, so using foam as the pillow content in the study can be as close as possible to the daily pillows of subjects and avoid the discomfort caused by pillow replacement. In addition, Li et al. [23] used buckwheat as a pillow content to imitate the subjects’ daily sleeping environment. In all the articles reviewed, only one study showed the mechanical parameters of pillow materials. Ren et al. [22] clearly pointed out that the Young’s modulus of the pillow and elevation mat used was 0.054 MPa and the Poisson’s ratio was 0.045 in their study on the effect of pillow height on the biomechanics of the head-neck complex. The material properties were measured using a material-testing machine. However, both foam and buckwheat are deformable materials that can be compressed in the experiment, and the actual height cannot be determined. Therefore, the experimental results may be misleading and unreliable in studies related to pillow height.

In terms of pillow shape, two studies designed a pillow that can maintain a normal head and neck posture mainly by measuring body dimensions. The newly designed pillow had a U-shaped front view. The pillow is lower in the middle in the supine position and higher in the lateral position on both sides [11,21]. Two studies used a typical B-shaped cervical pillow, which can support the cervical spine well and facilitate the measurement of pressure distribution and electromyographic activity in the neck [14,22]. A rectangular pillow was used in two other studies. This type of pillow is still widely used at present; thus, it is closer to the actual sleeping environment of participants [19,23]. Except for Ren et al. [22], the remaining articles did not describe the manufacturer, brand, and stiffness of the pillow samples.

### 3.3. Postures

Most people have their own habitual sleep posture; supine and lateral positions are the most common [27]. In the articles reviewed, supine and lateral positions were also the focus of the study. Generally, posture is controlled to ensure uniformity. For example, Kim et al. [20] prescribed a standard supine posture in which participants were instructed to place their heads resting on the pillow with the external occipital protuberance at the center of the pillow. Similarly, in the study by Ren et al. [22], the subjects were assisted in placing the external occipital protuberance of the skull on a reference point of the pressure mat, and the neck was placed on the highest point of the cervical pillow. The legs of the subjects were slightly separated, and the arms were placed at the sides of the body. In the work of Sacco et al. [19], the subjects were in the lateral position on a physical therapy treatment table; on the usual side, they typically slept with their hips and knees flexed to 90°. A pillow supported the knees, so that the hip and knees were aligned. Additionally, in another study, the posture was not controlled, and the sleep posture of the subjects was recorded by video. The duration and rotation frequency of the four sleep positions, including the supine, left lateral, right lateral, and prone positions, were analyzed to derive key points for the pillow design [21].

Sleep is a dynamic process, during which, individuals unconsciously change their sleep posture approximately 24 times per night to relieve fatigue, mainly in mutual conversions between the supine and lateral positions [21]. Studies have shown that the right sleep posture can improve the sleep quality. First, sleep posture affects the heart function. Gordon et al. [28] suggested that the diastolic, systolic, and mean arterial blood pressures were significantly lower in the lateral position than in the supine position. In addition, it was reported that the patients with congestive heart failure should avoid the left lateral position during sleep to prevent discomfort from an enlarged apical heart [29]. Second, sleep posture affects breathing. The supine position is more likely to cause asphyxia than the lateral position and aggravates the severity of sleep apnea [30,31]. The lateral position can lateralize the airway, reduce the incidence of airway collapse, and improve the airway expansion ability during sleep, effectively inhibiting the occurrence of sleep apnea [30,31]. Third, variations in sleep posture may produce different trunk bending angles and thus influence the spinal alignment [5]. The adoption of physiological spine curvature can reduce stress inside the spine, relax the muscles of the neck and back, and promote sleep. Therefore, sleep posture is a matter of significant concern.

Because the supine position is relatively simple, most of the current studies related to pillow height selected the supine position. The standard lateral position usually requires subjects to place their body perpendicular to the bed surface, but people tend to turn their shoulders forward toward the mattress [32]. Therefore, there are few studies on the lateral position owing to the difficulty of control. Prone and other intermediate postures were barely evaluated. In addition, most of the current studies only focused on the physiological and mechanical characteristics of the head, neck, and shoulder in the static posture, without analyzing the changing values of the parameters in the process of posture variation, which requires further investigation.

### 3.4. Determinants and Measurement Methods

**Figure 2:**
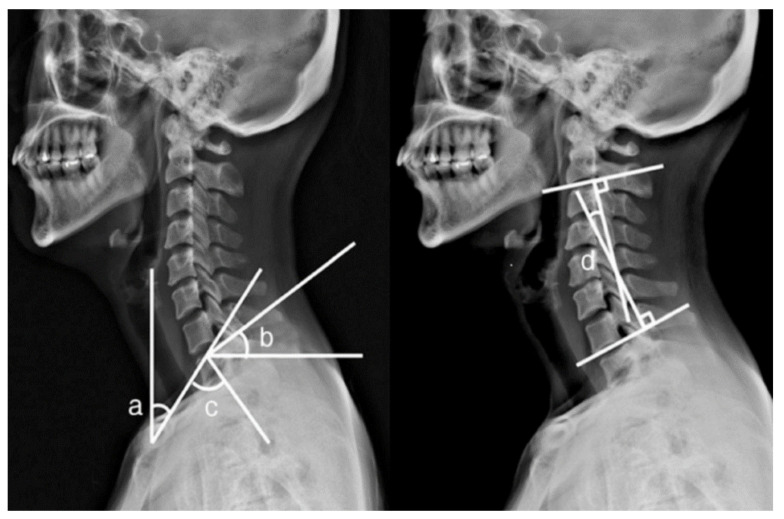
Parameters of cervicothoracic spine segments: (a) neck tilt; (b) T1 slope; (c) thoracic inlet angle; (d) C2-7 Cobb’s angle [20] (open access).

Cervical spine alignment or curvature is a very important factor, as the physiological curvature of the cervical spine can undertake the load of the head, reduce the external force concussion, and protect the spinal cord and brain. Maintaining the physiological curvature of the cervical spine is thought to prevent musculoskeletal problems or pain. It is generally believed that the physiological curvature of the cervical spine is maintained mainly based on endogenous and exogenous stability. Endogenous stability involves the vertebrae and ligaments, and is referred to as “static balance”. Exogenous stability involves the muscles and is referred to as “dynamic balance”. It is responsible for controlling and regulating the stability of the cervical spine. Both static and dynamic balances are used to maintain the physiological curvature and function of the cervical spine. Once there is a long-term imbalance in the endogenous and exogenous stabilities, some symptoms related to the cervical spine occur, such as numbness and weakness in the upper limbs, neck and arm pain, neck stiffness, headaches, and dizziness [33]. Among the articles reviewed, one measured the four morphological parameters of cervicothoracic spine segments under three pillow heights using radiographs, including neck tilt, T1 slope, thoracic inlet angle, and C2-7 Cobb’s angle (Figure 2) [20]. Finite element analysis is another commonly used method. Ren et al. [22] predicted cervical spine alignment under four pillow heights by constructing a finite element model for the head and neck. Three spine alignment parameters (cervical angle, lordosis distance, and kyphosis distance) were identified. In both the experiment and simulation, the measurements were performed by lying on the back.

**Figure 3:**
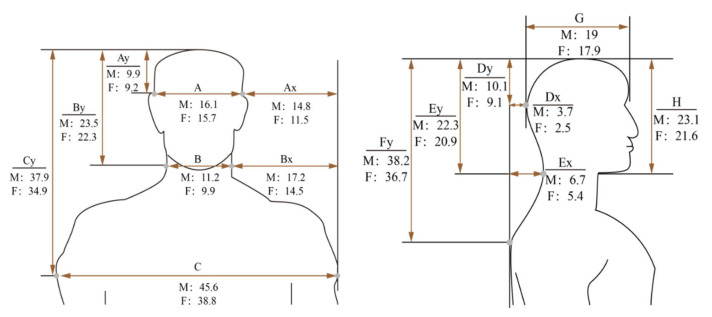
Anthropometric parameters measured by Cai and Chen [21]. Key parameters: width from ear to shoulder, Ax; the length from the hindbrain to wall, Dx; the length from neck to wall, Ex.

As a tool to support the head and neck, the pillow needs to have the right height to maintain the natural position of the head and neck. This requires the pillow height to be consistent with the body dimensions. Huang and Alice [13] measured the head girth, half shoulder length, and the distance of the external occipital protuberance to C7 using a whole-body 3D laser scanner. Two studies [11,21] measured body dimensions using a set of Martin’s anthropometric measuring instruments, including stature gauge, beam calipers, slide calipers, measure, and ruler. The key parameters included the width from ear to shoulder (height supporting the head in lateral position), length from hindbrain to wall (height supporting the head in supine position), and length from neck to wall (height supporting the neck in supine position) (Figure 3).

**Figure 4:**
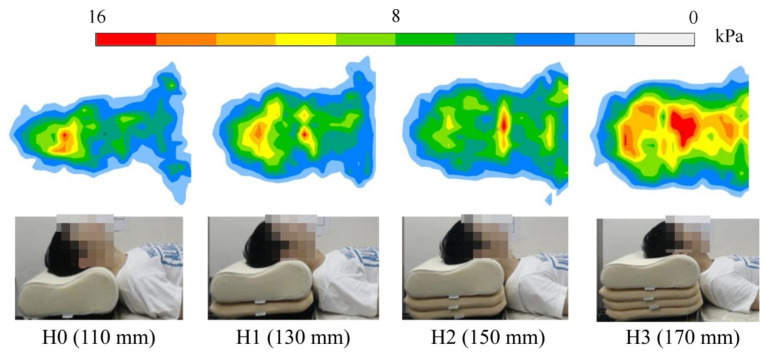
Cranial and cervical pressure distributions of a typical subject under four pillow heights [22] (open access).

Body–pillow contact pressure, which is related to sleep comfort, was the third most frequently investigated parameter [34]. Pressure induces the deformation of skin and thus triggers the sensation of touch (via mechanoreceptors) and pain (via nociceptors) upon high loading [5]. At the same time, high pressure may be poorly tolerated because fluid transfusion across soft tissues is affected by body contact pressure [35]. Therefore, body contact pressure is very important for sleep comfort and pain perception. The body pressure distribution is usually measured using thin and sensitive pressure sensors. Ren et al. [22] measured the pressure distribution of the cranial and cervical regions using a pressure-sensitive mat, which consists of 1024 sensors and can provide pressure data with a resolution of 32 × 32 points (Figure 4). Likewise, the American TEKscan body pressure measurement system (BPMS) was used to measure the pressure distribution of the head, neck, chest, waist, and hip in the work of Li et al. [23]. The BPMS TEKscan system is characterized by accuracy, high resolution, fast sampling, and non-invasiveness, and is widely used by medical practitioners and researchers to assess numerous health issues, especially those related to foot, gait, posture, rehabilitation, and ergonomics. The common measurement parameters related to the pressure distribution include the average pressure, peak pressure, and contact area. The above parameters in different regions of the body are often investigated and analyzed. In addition, Li et al. [2] calculated the pressure gradient, which is the rate of change in pressure along a certain direction. This reflects the degree of difference between the adjacent pressures.

**Figure 5:**
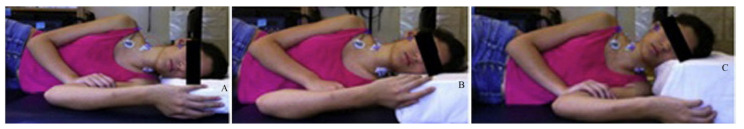
Electromyographic activity measurements of neck and shoulder muscles under three different pillow heights: 5 cm (**A**), 10 cm (**B**), and 14 cm (**C**) [19].

Muscle activity is also an important indicator for evaluating whether pillow height is appropriate. It was demonstrated that pain and discomfort were related to higher and longer trapezius EMG activity in participants with neck and shoulder pain [36]. In another study, neck pain was found to cause substantial muscle inhibition in bilateral elbow flexors [37]. Therefore, abnormal cervical muscle activity may be a potential cause of neck pain and discomfort [38]. The muscle activity measurement system was characterized using circular electrodes. Sacco et al. [19] used an EMG system for the measurement, including an analogic-to-digital converter with 16 bits of resolution with a signal amplification factor of 2000 and disposable Ag/AgCl circular electrodes. Ag/AgCl circular electrodes were placed on the muscles of the neck and shoulder to acquire data, and an analogic-to-digital converter was used for data processing (Figure 5). Similarly, in the work of Wang et al. [14], extensor digitorum communis muscle activity was recorded using silver/silver chloride surface electrodes. Electromyography is often performed in the sternocleidomastoid, upper trapezius, and middle trapezius [19,24]. In addition, muscle strength of the upper extremity muscle has also been measured, such as the extensor digitorum communis muscle [14].

Cervical spine alignment, body dimension, contact pressure, and muscle activity are quantitative and objective indicators, which are determinants of pillow height evaluation. However, to ensure the integrity of the study, some subjective evaluation methods should also be considered. Common subjective evaluation methods include questionnaires and subjective comfort evaluations. Questionnaires mainly set up sleep-related questions for subjects to answer after a period of sleep. Common questions include sleep duration, waking pain, sleep apnea, and the number of times to wake up [13,21,25]. Subjective comfort evaluation is usually carried out using a visual analog scale (VAS). For example, Sacco et al. [19] evaluated the perceived comfort in the neck, shoulders, and upper trunk for each pillow height using a 100 mm VAS (0 mm as the less comfortable and 100 mm as the most comfortable). Subjective evaluations are easily affected by individual perceptions. Additionally, time is an important factor that affects subjective comfort. Some patients may feel uncomfortable when they first use the neck pillow, but gradually accept it after long-term use [39]. This indicates that the acceptance of new things requires an adaptation time. In terms of pillow height, a habitual pillow height is comfortable, but may cause the cervical spine to be in an incorrect position. Therefore, subjective evaluation is misleading and unreliable, which may lead to an inappropriate choice of pillow height that induces or worsens neck pain. Although subjective evaluation may not be used as a determinant of the appropriate pillow height, it can play a certain reference role in assessing the comfort of a certain pillow height.

### 3.5. Optimization or Selection Criteria

Cervical spine alignment, body dimension, contact pressure, and muscle activity are usually measured to explore the effect of pillow height on the physiological and mechanical characteristics of the head-neck-shoulder complex, which are the predominant parameters of pillow height design and optimization. As the essence of body dimension measurement is to provide the pillow with the right height to support the head and neck and maintain the physiological curvature of the spine, body dimension measurement can be classified as spinal alignment.

It is well accepted that spinal alignment in an upright posture is optimal. In the supine position, the spine needs to be S-shaped in the sagittal plane, while in the lateral position, the spine needs to be horizontal in the coronal plane. In this way, the head and neck can be in the middle, aligned with the spinal line of the mid-upper back and orthogonal to the shoulder line, thereby minimizing biomechanical stress in these regions and maintaining muscle balance during sleep [10,40]. However, Verhaert et al. [32] stated that lumbar lordosis increases when standing under the influence of gravity, while it tends to be flattened when lying. Therefore, it is not appropriate in theory to consider upright posture as the optimal spinal alignment for sleep.

Corresponding to the spinal alignment, some researchers believe that pillow height should be consistent with body dimensions, and pillow shape should match the bony structure of the head, neck, and shoulder to maintain the correct posture of the head and neck [11,21]. However, Erfanian et al. [41] found no statistically significant correlation between anthropometric parameters and pillow height preference, which was confirmed by Wang et al. [14]. Therefore, they believed that the body dimensions of an individual may not serve as a good predictor of an appropriate pillow height.

As high pressure can affect the blood circulation of the subcutaneous tissue and lead to numbness and pain, efforts have been made to reduce the body-mattress peak pressure and achieve a more even pressure distribution as early as the mattress design process [42,43]. However, high pressure also means sufficient support, so the pressure should not be too high or too low. It was shown that different body regions can exhibit different pressure tolerances [44]. Accordingly, the contact pressure between the pillow and body should be sufficiently high to provide sufficient support to the body, but should not exceed the tolerance thresholds for various parts of the body. Pillows with different stiffness values in the head and neck regions are expected to achieve this goal.

The main function of the musculoskeletal system during sleep is to support the weight of the body, thereby allowing the muscles and intervertebral disks to recover from an almost continuous load throughout the day [45]. To achieve this, sleep posture requires bilateral symmetrical muscle activity and minimal electrical activity. Thus, better comfort may be associated with lower muscle activity resulting from better alignment of the head and shoulders and symmetry between the body sides [19]. In addition, Wang et al. [14] revealed that maximal muscle strength is related to the best comfort, which may be a useful complement for selecting the optimal pillow size. 

In conclusion, the optimal pillow height should maintain the physiological curvature of the cervical spine during sleep, which needs to conform to the anthropometric parameters of the head and neck. In addition, the optimal pillow height should provide sufficient support for the head and neck. Simultaneously, the stress should not exceed the tolerance threshold of the body. Finally, the influence of pillow height on the electromyographic activity of neck and shoulder muscles also needs to be considered. When the electromyographic activity was low, the muscle strength was large, and the height was more suitable. To date, the optimal range of these parameters has not been proposed to achieve better spine alignment and comfort. It is important to clarify the ideal range of these parameters to provide guidance for ergonomic pillow design and pillow selection in daily life.

### 3.6. Study Scope and Key Findings

In the reviewed articles, two studies designed a suitable pillow for both supine and lateral positions to promote sleep quality [11,21]. They proposed the key points for pillow design: (1) The pillow height for the supine and lateral positions should be different. (2) The pillow height for men and women should also be different. (3) Pillows should be raised in the neck region to support the cervical spine. According to the results of body dimension measurements, pillow prototypes for males and females were made of foam rubber. The basic form of the pillow for both genders is a U-shaped front view. The pillow is lower in the middle in the supine positions and higher in the lateral position on the two sides. One study constructed an ideal head and neck support model based on knowledge of the ideal body pressure distribution matrix, partition body pressure distribution indicators, and pressure sensitivity weights [2]. Two articles used linear regression analysis to establish the relationship between the optimal pillow height and anthropometric parameters, and optimal pillow height and contact pressure [13,23].

Five articles aimed to develop recommendations for the optimal selection of pillow height. These studies reported the effects of different pillow heights on cervical spine alignment, body–pillow contact pressure, and muscle activity [19,20,22,23,24]. First, pillow height has an important influence on the physiological curvature of the cervical spine during sleep. As the height of the pillow increased, the T1 slope and C2-7 Cobb’s angle increased [20]. That is, a pillow that is too high can cause the cervical spine to bend forward, whereas a pillow that is too low can cause the cervical spine to stretch backwards. Both conditions are not conducive to the maintenance of cervical physiological curvature and may induce stress imbalance within the cervical spine. Second, pillow height also has an important impact on the pressure distribution of the body during sleep. Pillow height elevation significantly increased the average and peak pressures in the cranial and cervical regions [22]. Moreover, the results of Li et al. [23] showed that the peak contact pressure gradually shifted from the head to the hip area as the pillow height increased. The support center of the pillow dynamically changes with the pillow height, and the load-bearing ratios of the head, neck, and shoulder regions are redistributed [22]. Therefore, the pressure distribution is an indicator for evaluating the appropriateness and comfort of pillow heights. Finally, pillow height affects muscle activity in the neck and mid-upper back. Li and Huang [24] indicated that the muscle activities of the sternocleidomastoid decreased mostly when using a neck support pillow and provided a relaxation condition. The condition without pillows can cause an unstable posture and keep the sternocleidomastoid activated to maintain craniopostures. In the work of Sacco et al. [19], a pillow height of 10 cm elicited the lowest EMG activity of the neck and upper trunk and the best comfort. The highest electrical activation was at a pillow height of 5 cm, followed by a pillow height of 14 cm. In view of the above, it is considered essential to use electromyographic activity as another indicator of pillow height fitness for healthy sleep. In addition, one study revealed that the mechanism of extensor digitorum communis muscle strength changes with different pillow size preferences. The authors believed that altered afferent input of the cervical spine caused by uncomfortable pillows results in reflex-type muscle inhibition of the upper extremity.

Different studies have different opinions on the optimal pillow height in the supine and lateral positions. Kim et al. [20] recommended that the most suitable pillow height is 10 cm in the supine position, considering normal cervical lordosis. Li et al. [23] regarded 7 cm as the most comfortable height for the supine position. The results of Sacco et al. [19] showed that 10 cm represents the best comfort for the lateral position. Thus, there remain no firm conclusions about the optimal pillow height for the supine and lateral positions.

## 4. Discussion

Neck symptoms and sleep problems have attracted increasing attention, and individuals have begun to acknowledge the importance of daily sleep and pillows more seriously. Pillow height can gravely affect the cervical spine alignment, and thus should be given priority in pillow choice. Many hypotheses have been proposed regarding the optimal pillow height, but there are no firm conclusions. Herein, we summarize the research progress on pillow height and analyze the effects of pillow height on the physiological and mechanical characteristics of the head-neck-shoulder complex. Several determinants for the appropriate pillow height have been proposed, including cervical spine alignment, body dimensions, contact pressure, and muscle activity. The measurement methods and key findings of each parameter were described in detail.

Cervical spine alignment reflects the complex biomechanical interaction between the body and the pillow during sleep, which is an important physiological characteristic of the neck. Current research methods mainly include radiography and finite element analysis. The parameters correlated with cervical sagittal balance are usually measured, such as T1 slope (T1S). Although it was demonstrated that patients with a T1S value between 13° and 25° mostly had better sagittal balance than patients with values outside this range, its occurrence does not guarantee normal sagittal balance [46]. To date, no studies have established normative sagittal T1S values. The coronal parameters of the cervical spine associated with the lateral position have not been studied under different pillow heights. Furthermore, it is theoretically inappropriate to regard upright spine curvature as the desired alignment because spine loading modes are completely different when standing and lying down [5].

The pillow shape needs to match the bony structure of the head and neck to maintain the physiological curvature of the cervical spine. Contour pillows such as U-shaped pillows and B-shaped pillows are very popular in the market. The key to contour pillow design is that the pillow height in the cranial and cervical regions needs to be consistent with the body dimensions. Erfanian et al. [41] and Wang et al. [14] proposed no statistically significant relationships between body dimensions and pillow height preference, which was contrary to the results of the study conducted by Chen and Cai [11,21]. This may be due to the differences in their measurement parameters. The anthropometric parameters included the body mass index (BMI), neck length, and neck width in the work of Wang et al. [14]. The body dimensions related to the pillow height for the supine and lateral positions are the width from the ear to the shoulder, the length from the hindbrain to the wall, and the length from the neck to the wall [11,21]. These parameters allow the pillow to provide sufficient support for the head and neck to maintain the natural curvature of the cervical spine. The pillow prototype, designed according to body dimensions, is usually made from soft materials such as foam. When lying on the pillow prototype, the head and neck exert pressure on the pillow and the pillow deforms, so that the pillow height is not sufficient to maintain the cervical spine alignment. Therefore, increasing the pillow height at the head and neck should be considered to compensate for the height loss of the pillow after compression.

**Figure 6:**
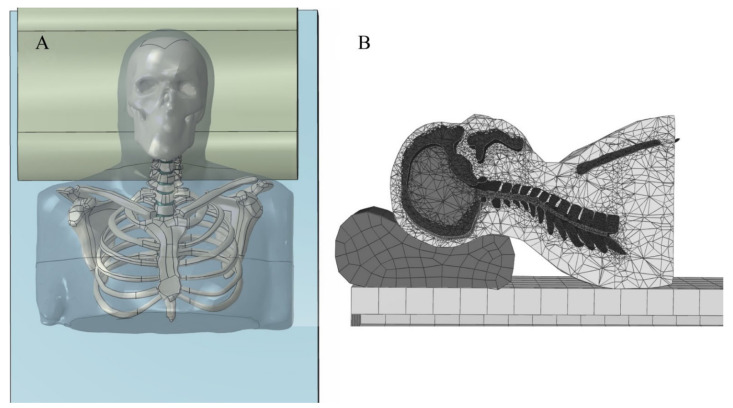
Finite element model of head and neck: (**A**) frontal view; (**B**) side view [22] (open access).

The contact pressure reflects the internal mechanical characteristics of the head and neck during sleep, which is the gold standard for pillow height evaluation. To date, the contact pressure is often measured by pressure sensors. In addition to the experimental methods, finite element analysis may provide tangible results. The three-dimensional finite element model of the head and neck constructed from the CT images of healthy individuals can be used to simulate and analyze the stress characteristics of the cervical spine under different sleep postures and pillow heights (Figure 6) [22]. However, because of the simplification of the model, the influences of muscles and ligaments on cervical spine movement are generally not considered in the simulation process. To date, studies on body–pillow contact pressure under different pillow heights remain immature. Different body regions feature different pressure sensitivities and tolerances [44]. High pressure indicates that the pillow can provide adequate support, but may be poorly tolerated because the fluid transfusion across soft tissues is affected by the body contact pressure [35]. Studies have shown that capillaries can better perform perfusion into tissues when the skin contact pressure is lower than 4.2 kPa; this value also represents better comfort [47]. Accordingly, the body–pillow contact pressure should be high enough to support the head and neck, but should not exceed the tolerance thresholds for the various parts of the body. In this field, proposing an optimal range of biomechanical parameters for achieving both a suitable supporting force and comfort is crucial.

**Figure 7:**
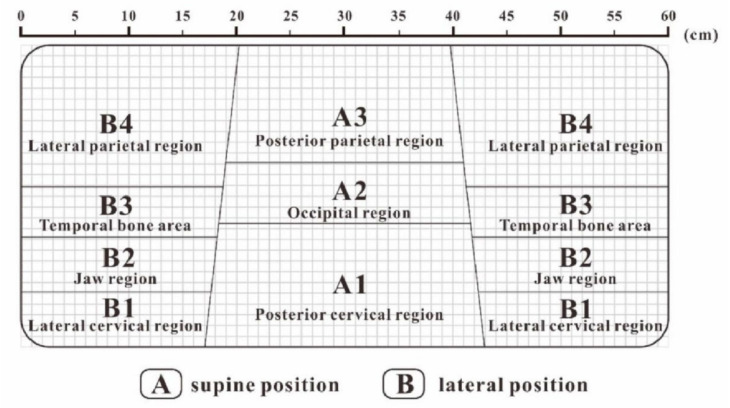
Pillow partition: three partitions for the supine position (A1, A2, A3) and four partitions for the lateral position (B1, B2, B3, B4) [2] (open access).

**Figure 8:**
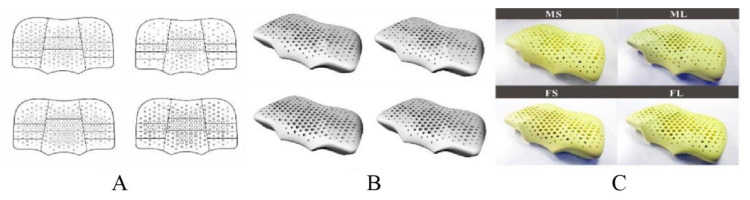
Pillow prototypes made of pore-array structure with different apertures: (**A**) partition and array; (**B**) three-dimensional model; (**C**) standard pillow prototype [2] (open access).

The work of Li et al. [2] provided a novel solution for an ideal head and neck support model. First, the ideal pressure distribution matrix can be obtained by calculating the average pressure distribution matrix of several samples with the highest comfort score. Second, the ideal pressure distribution matrix can be divided by the fuzzy clustering algorithm into three partitions for the supine position and four partitions for the lateral position, which can be mapped to the corresponding regions of the pillow (Figure 7). The ideal body pressure distribution index for each partition was also calculated. Third, the analytic hierarchy process based on expert evaluation of head and facial tissues can determine the pressure sensitivity weight of each partition. Finally, an ideal head and neck support model can be constructed based on knowledge of the ideal body pressure distribution matrix, partition body pressure distribution indicators, and pressure sensitivity weights. Because different body regions require different supports, the pillow prototypes must have different stiffness in each partition. To achieve this, Li et al. [2] fabricated a pore-array structure with different apertures on the material to obtain different elastic coefficient changes under the same volume of material, which guides the development of ergonomic pillows (Figure 8). The weakness is that the ideal pressure distribution matrix was determined according to the pressure distribution matrix of the pillows with a higher subjective comfort evaluation, which was not analyzed in combination with objective physiological indicators, such as the pressure of normal blood perfusion and uncompressed nerve. Therefore, the establishment of an objective and reasonable range of stress for different body regions has become a popular research topic and is expected to remain an important development trend in the future.

Muscle activity usually represents the physiological characteristics of the neck and shoulder muscles. An appropriate pillow height promotes better alignment of the head, neck, and shoulders, as well as symmetry between the body sides, which can reduce muscle activity and produce a better perception of comfort [19]. Therefore, we speculate that the decrease in muscle activity may be related to good skeletal support. To date, muscle activity is mainly measured using electrodes. Other measurement methods have not yet been reported and need to be explored and developed. In addition, most studies have evaluated skin surface electromyography values, but it is difficult to obtain the expected results owing to the complex muscle locations [14].

A comprehensive evaluation combining the above determinants provides a unique solution for ergonomic pillow design and proper pillow height selection. However, compromising and weighing multiple determinants to establish a scientific and standard evaluation method for pillow height remains difficult. At the same time, the optimal range of these determinants to realize design optimization and high-quality pillows has not been proposed. Further exploration of these aspects is required.

This study has several limitations. First, we did not assess the quality of the articles reviewed, as the involved study designs and scopes were diverse and thus difficult to compare. Second, the majority of the articles reviewed mainly concentrated on short-term testing procedures, which did not assess the long-term effects of pillow use. Third, eligible articles were determined by several manual screening processes and discussions, so the repeatability of the search and screening may be challenged. Finally, this paper only aims at ordinary individuals, without considering pilots, astronauts, and other special populations. Studies have shown that astronauts are at increased risk of cervical and lumbar disk herniation and often have symptoms of back pain, also known as “space adaptation back pain” [48]. Back pain, which mostly occurs in the early stages of space flight, is one of the most common musculoskeletal syndromes in space. The exact mechanism is unclear owing to the lack of in-orbit imaging capabilities. However, it can be inferred from the increase in spinal length during space flight that these symptoms are caused by disk swelling owing to the microgravity environment in space [49]. Likewise, pilots often suffer from cervical, lumbar, and limb injuries owing to repeated overweighting and weightlessness, as well as vibration loads that occur during flight training. To date, common countermeasures against such syndromes for pilots and astronauts include stretching, exercise, and physical training. A customized pillow with an appropriate height should be regarded as a complement to physical therapy to relieve the symptoms of the neck and back in astronauts and pilots during sleep, which is crucial for the health and safety of astronauts and pilots, as well as for the successful implementation of their flight missions.

## 5. Perspective

Pillows with a uniform height, which are common in the market, are not suitable for both supine and lateral positions during sleep. Consequently, Chen and Cai [11] designed a U-shaped pillow, which is lower in the middle area for the supine position and higher on both sides for the lateral position. However, sleep is a dynamic process during which individuals unconsciously change their sleep posture approximately 24 times per night [21]. The design of the U-shaped pillow may not ensure that people just lie in the corresponding sleeping region of the pillow in a certain sleeping posture. Therefore, a smart pillow that can recognize the sleeping posture and automatically adjust its height according to the sleeping posture needs to be developed and put into the market. With the rapid developments in the Internet of Things, big data, and artificial intelligence, the realization of smart pillows is possible. For example, Zhang et al. [50] developed a smartphone-based automatic pillow system for detecting and treating sleep apnea. The system included built-in blood oxygen sensors for detecting sleep apnea events in real time and automatically adjusting the height and shape of the pillow to stop a sleep apnea event. Yang [51] developed a pillow that adjusts the pillow height of the neck area. The outer layer was soft and fluffy cotton, and the inside was filled with air through a rubber hose. A built-in sensor can detect the weight of the user’s head and neck. The Bluetooth sensor of the smartphone and a pillowcase detection sensor are interlocked, and the pillow height can be customized according to the information collected in combination with the smartphone application, realizing free adjustment of the pillow height. Although these pillows allow for height freedom, it is still unclear whether the height after adjusting can meet the users’ dual needs of comfort and health.

Changing the bad habits of the users when using pillows is a step-by-step process. The comfort perception is related to time, so a significant change in the pillow height may cause discomfort due to the user’s inability to adapt [52]. Dynamic adaptation to the pillow height refers to a staged fine-tuning of the pillow height, which can correct the cervical spine step-by-step, based on a small adjustment in the pillow height instead of a large change. Users can better adapt to the pillow height and subtly improve their bad habits pertaining to the use of pillows, thereby achieving a balance between comfort and health. In the future, smart pillows will eventually have the capability to automatically measure the user’s anthropometric parameters, record the user’s habits when using pillows, and collect the biomechanical data of the head and neck with real-time monitoring. An optimal pillow height setting scheme for different sleep postures is recommended for users through big data analyses. Eventually, the smart pillow will be able to automatically fine-tune the pillow height according to the setting scheme to gradually improve the user’s cervical curvature and sleep quality.

## 6. Conclusions

In view of the limitations of the current pillow height evaluation studies, we summarized the research progress in this field and proposed several quantitative and objective indicators for pillow height evaluation, including cervical spine alignment, body dimension, contact pressure, and muscle activity. Future studies need to focus on the ideal range of these parameters to achieve optimal pillow comfort, and a reasonable algorithm must be developed to weigh multiple determinants. It is expected that in the future, smart pillows with automatic height adjustment will improve sleep health better than traditional pillows with fixed heights, and will have a huge potential market.

## Figures and Tables

**Table 1:** Selection of subjects and pillow samples in the reviewed articles.

Author (Year)	Subjects	Exclusion Criteria	Pillow Characteristics	Manufacturer	Sleeping Postures
**Spinal alignment**					
Kim et al. (2015) [20]	16 asymptomatic adult subjects Gender: NA Age: 20–30 years	Spinal diagnosis, symptoms, or treatment; Accident or injury to the cervicothoracic spine in the preceding year; Currently receiving treatment for neck symptoms.	NA	NA	Supine
**Body dimension measurements**					
Huang & Alice. (2007) [13]	40 healthy adult subjects Gender: 20 males, 20 females Age: NA	Organic pathology, neck pain.	A urethane foam foundation, an overlying “memory foam” supporting the head and neck, a stretch terry-cloth cover, and a cotton pillowcase.	NA	No control
Chen & Cai. (2012) [11]	20 students Gender: 10 males, 10 females Mean age: 22.8 ± 1.3 years	NS	NA	NA	No sleeping, the body dimensions of participants were measured in the standard standing posture.
Cai & Chen. (2016) [21]	40 healthy subjects Gender: 20 males, 20 females Mean age: 25.7 ± 7.1 years 40 healthy subjects Gender: 20 males, 20 females Mean age: 34.4 ± 13.0 years 6 healthy subjects Gender: 3 males, 3 females Mean age: 34.2 ± 4.1 years	Sleep disorder; Have important work to do the next day. No physical impairments. Sleep disorder; Have important job the following day.	NA NA Materials: foam rubber Pillow types: new designed pillow prototype and current pillow of participants.	NA NA NA	A three-step testing procedure: No control, sleeping postures were recorded for further analysis. No sleeping, the body dimension of participants were measured in the standard standing posture. No control, the participants were asked to sleep using their ordinary sleeping postures.
**Pressure distribution in the head and neck**					
Ren et al. (2016) [22]	10 healthy subjects Gender: 5 males, 5 females Mean age: 26 ± 3.6 years	Chronic myofascial pain, acute injury, or inflection over the spine.	Materials: polyurethane foamShape: B-shaped cervical pillow	Benelife, Infinitus Co. Ltd., China	Supine
Li, Hu & Liao. (2018) [23]	19 quinquagenarian women Gender: 19 females Mean age: 53.74 ± 6.80 years	Cervical/lumbar disease; Cannot complete the questionnaire independently.	Materials: buckwheatSize: 55 × 35 cm	NA	Supine
Li et al. (2021) [2]	6 graduate students Gender: males and females, the number is not specified. Mean age: 25 ± 2 years	Abnormal cervical curvatures; Unhealthy bodies.	7 common ergonomic sleeping pillows with different size on the market. Materials: memory foam Density: 60D or 40D	NA	Supine and lateral
**Electromyographic activity of neck and shoulder muscles**					
Lin & Huang (2007) [24]	30 young volunteers Gender: NA Mean age: NA	Neck disability	NA	NA	Supine
Sacco et al. (2015) [19]	21 young volunteers Gender: 6 males, 15 females Mean age: 24.3 ± 6.7 years	Neurologic cervicalgia, temporomandibular disorders, cervical disc disease, sleep disorders, or shoulder joint lesions.	Materials: foam Shape: a rectangular shape Size: 55 cm width	NA	Lateral
Wang et al. (2015) [14]	Healthy individuals Age: >18 years	Injury to the cervicothoracic spine or dominant upper limb in the previous 6 months; Have a neurological or orthopedic condition on the dominant upper limb, cervicogenic dizziness/ headache; Currently receiving treatment for cervicothoracic spine pain.	Eleven cervical pillows with the same content but different sizes were tested. The pillow height varied from 6 cm to 12 cm and pillow width from 31 cm to 34 cm. The length of the pillows was 61 cm.	NS (from the same manufacturer)	Supine

Notes: NA, not accessible; NS, not specified.

**Table 2:** Measurement methods and outcomes of parameters in the reviewed articles.

Author (Year)	Measurement	Instrument (Methods)	Manufacturer	Interventions	Measurement Outcome
**Spinal alignment**					
Kim et al. (2015) [20]	Parameters of Cervicothoracic Spine Segments	Radiographs	NA	All participants were asked to try three pillows of different heights (0, 10, 20 cm).	The thoracic inlet angle (TIA), T1 slope (T1S), neck tilt (NT), and C2-7 Cobb’s angle.
**Body dimension measurements**					
Huang & Alice. (2007) [13]	7-Day Daily Sleep Log Assessment of sleep quality Anthropometric parameters Pressure distribution	Questionnaire, record the time associated with sleep. Sleep Quality Visual Analogue Scale (SQVAS): It can assess individual subjective feelings of overall sleep quality with a possible score ranging from 0 to 10. Whole Body 3D Laser Scanner FSA pressure mapping system	NA NA From Chang Gung University, Taipei, Taiwan NA	NI NI NI NI	Time to go to bed, time to fall sleep, time to wake up and so on. Score for sleep quality. Body weight, head girth, half shoulder length, the distance of external occipital protuberance to cervical seven. Physiological interface pressure imaging (NS)
Chen & Cai. (2012) [11]	Body dimension	A set of Martin’s anthropometric measuring instrument: stature gauge, beam calipers, slide calipers, measure, and ruler; Body-curve measurer.	NA;Made by the study.	NI	Some body dimensions, mainly including: the width from ear to shoulder, the width from neck to shoulder, the length from hindbrain to wall, the length from neck to wall.
Cai & Chen. (2016) [21]	Sleep position investigation Body dimension measurements Sleep quality test	Infrared video Automatic stature and weight scale; the Martin’s anthropometric measuring instruments, including stature gauge, beam calipers, slide calipers, outside calipers, tape measure, and ruler. A compact sleep quality recorder called EZsleep (TX-EK3).	NA NA DynaDx Corporation	NI NI All participants were asked to sleep on the new designed pillow prototype and their currently used pillows for three days each.	Sleep positions were classified into four types: supine position, left lateral position, right lateral position, and prone position. The duration, rotation frequencies and the proportion of duration of the four sleep positions were recorded. Some body dimensions, mainly including: the width from ear to shoulder, the width from neck to shoulder, the length from hindbrain to wall, the length from neck to wall. Sleep quality indexes, such as total sleepduration, time taken to fall asleep, sleep apnea and so on.
**Pressure distribution in the head and neck**					
Ren et al. (2016) [22]	Craniocervical pressure distribution Cervical spine alignment	Pressure sensitive mat Abaqus finite element model	BodiTrak BT1526, Vista Medical Ltd., Winnipeg, MB, Canada Mimics (Materialise, Leuven, Belgium); Abaqus (Dassault Systèmes, RI, USA).	All participants were asked to try four pillowsof different heights (11, 13, 15 and 17 cm). Finite element predicted position of the cervical vertebrae under the four pillow height conditions.	Cranial region: average and peak pressure;Cervical region: average and peak pressure. CA: Cervical Angle; LD: Lordotic distance; KD: Kyphotic distance.
Li, Hu & Liao. (2018) [23]	Subjective Comfort Evaluation Body Pressure Distribution	The score ranged from 0 to 5, and 5 is the full score. Subjects were asked to rate the comfort of the following body parts: head, shoulder, waist and hip. BPMS TEK scan system	NA NA	NI All participants were asked to try four pillows of different heights (3, 7, 11 and 15 cm).	Head, shoulder, waist and hip comfort score Peak Force, Peak Contact Pressure, Contact Area on the following body parts: head, shoulder, waist and hip.
Li et al. (2021) [2]	Body Pressure Distribution Subjective Comfort Evaluation Body dimension	American Tekscan body pressure measurement system (BPMS) Evaluation form, including four items: softness, wrapping, support, and fit. Each item was scored on a 5-point scale, with 1 being the least comfortable and 5 being the most comfortable A profile ruler and a Martin measurement instrument.	NA NA NA	All participants were asked to try 7 common ergonomic sleeping pillows with different size on the market. NI NI	Average pressure, peak pressure, maximum pressure gradient, and average pressure gradient Score for comfort Anthropometric parameters of head, neck and shoulder.
**Electromyographic activity of neck and shoulder muscles**					
Lin & Huang (2007) [24]	Craniocervical postures Neck muscle activities	Universal goniometer. NA	NA NA	Interventions included different pillow conditions and time. (NS) All participants were asked to try three conditions: a neck support pillow, a standard pillow, and without using pillow.	The craniocervical angle in sagittal, frontal, transverse plane. Electromyography (EMG) of sternocleidomastoid (SCM) and upper trapezius (UT).
Sacco et al. (2015) [19]	Comfort evaluation. Electromyographic (EMG) activity of the neck and mid-upper back	100-mm visual analog scale (VAS; 0 mm as the less comfort and 100 mm being the most comfortable). EMG system, including: an analogic-to-digital converter and Ag/AgCl circular electrodes	NA Model 800C; EMG System do Brasil, São José dos Campos, Brazil	NI All participants were asked to try three pillows of different heights (5, 10, 14 cm).	Comfort score in the neck, shoulders, and upper trunk for each pillow height Electromyographic (EMG) activity of upper trapezius, middle trapezius, sternocleidomastoid muscle.
Wang et al. (2015) [14]	Subjective comfort evaluation Anthropometric parameters Extensor digitorum communis (EDC) muscle activity	Subjects experienced on pillows and verbally described comfort. NA Silver/silverchloride surface electrodes	NA NA Model 9013S0242, Alpine Biomed, Skovlunde, Denmark	NI NI All participants were asked to try four pillow conditions: most comfortable pillow, next most comfortable pillow, next worst pillow and worst pillow.	Pillows are classified as most comfortable pillow, next most comfortable pillow, next worst pillow and worst pillow. Body mass index (BMI), neck length, and neck width. The isometric maximal voluntary contraction force and surface EMG of the extensor digitorum communis (EDC).

Notes: NA, not accessible; NS, not specified; NI, no interventions.

**Table 3:** Study scope and key findings of the reviewed articles.

Author (Year)	Study Design	Scope/Objective	Key Findings
**Spinal alignment**			
Kim et al. (2015) [20]	Crossover design	To investigate the effect of different pillow heights on the slope of the cervicothoracic spine segments.	As the height of the pillow increased, the T1S and C2-7 Cobb’s angle increased, while the NT values tended to decrease. The TIA values, however, remained constant. The 10 cm is recommended as the most suitable pillow height to maintain the physiological curvature of cervical spine.
**Body dimension measurements**			
Huang & Alice. (2007) [13]	Repeated measurements	To find out the optimal pillow height fit for comfortable sleep.	A linear regression equation between pillow height and anthropometric parameters was established.
Chen & Cai. (2012) [11]	Repeated measurements	To determine the pillow dimensions for fitting supine and lateral positions for Taiwanese.	Two pillow models for male and female were designed. The basic form of the pillow for both genders is a U form from the front view. The pillow is lower in the middle for supine position and higher on the two sides for lateral position. A neck rest with 1.5 cm of height is proposed to pillow design for neck support during sleep. As a result of the difference of male and female body dimensions, the pillow size for female and male is also different. For male, the form of the base of pillow is a rectangle with a width of 75 cm and a depth of 40 cm from the top view. The height of middle area and both side are 4 cm and 14 cm, respectively. For female, the form of the base of pillow is a rectangle with a width of 70 cm and a depth of 35 cm from the top view. The height of middle area and both side are 2 cm and 12 cm, respectively.
Cai & Chen. (2016) [21]	Repeated measurements	Design of a suitable pillow for promoting sleep quality based on: Sleep position investigation to derive key-points for a pillow design. Body dimension measurements to determine pillow sizes. Pillow concept design to create pillow prototype. Sleep quality test to evaluate the new pillow prototype and the current pillow.	The supine and lateral positions were alternatively 24 times a night, and the current pillows were too high for the supine position and too low for lateral positions. The pillow height was quite different in supine position and lateral position and needed to take into consideration for a pillow design. In addition, the pillow height was also different for male and female, which was related to their body dimensions. A neck rest should be considered for neck support in the supine position. The pillow prototype was a U-form in the front of view. The pillow height in the middle area was lower for the supine position, and both sides were higher for the lateral position. The newly designed pillow led to significantly higher sleep quality, and the new design received an innovation patent.
**Pressure distribution in the head and neck**			
Ren et al. (2016) [22]	Randomized crossover trial, validation of simulation	To evaluate the effect of pillow height on craniocervical pressure and cervical spine alignment.	Craniocervical pressure: The average cranial pressure at pillow height H3 was approximately 30% higher than that at H0, and significantly different from those at H1 and H2 (*p* < 0.05). The average cervical pressure at pillow height H0 was 65% lower than that at H3, and significantly different from those at H1 and H2 (*p* < 0.05). The peak cervical pressures at pillow heights H2 and H3 were significantly different from that at H0 (*p* < 0.05). Cervical spine alignment: Raising pillow height from H0 to H3 caused an increase of 66.4% and 25.1% in cervical angle and lordosis distance, respectively, and a reduction of 43.4% in kyphosis distance.(H0: 11 cm; H1: 13 cm; H2: 15 cm; H3: 17 cm)
Li, Hu & Liao. (2018) [23]	Crossover design	To explore the effect of different height buckwheat pillows on body pressure distribution and comfort for quinquagenarian women.	As the pillow height increased, neck peak contact pressure, contact area and peak force increased. At the same time, the peak force and peak contact pressure gradually shifted from the head to the hip area. It has shown that 7cm pillow height was more comfortable for supine position compared to the rest heights in this study.
Li et al. (2021) [2]	Crossover design	To construct an ideal pressure distribution model for head and neck support through research on the partitioned support surface of a pillow.	An ideal support model with seven partitions, including three partitions in the supine position and four partitions in the lateral position, was constructed. The ideal body pressure distribution matrix and ideal body pressure indicators and pressure sensitivity weights for each partition were provided. The pillow that was designed and manufactured based on this model reproduced the ideal pressure distribution matrix evaluated by various groups of people.
**Electromyographic activity of neck and shoulder muscles**			
Lin & Huang (2007) [24]	Randomized crossover trial	To examine the changes of neck muscle activities when using different kinds of pillow.	The neck muscle activities of sternocleidomastoid was decreased mostly when using neck support pillow and been a relaxation condition. The condition without pillows would cause an unstable posture and keep sternocleidomastoid activated to maintain cephalic postures. The neck muscle activities of trapezius both had not changed within 30 min in supine position no matter what conditions with pillows or not were used.
Sacco et al. (2015) [19]	Single-blind randomized crossover trial	To evaluate the comfort and the electromyographic (EMG) activity of the neck and mid-upper back of asymptomatic adults using foam pillows of 3 different heights.	EMG activity: The middle trapezius muscle of the down-side showed the highest EMG activity in height 1 when compared with heights 2 (*P* = 0.0163) and 3 (*P* = 0.0313), with no statistical significance between pillow heights 2 and 3 for this muscle. There were no statistical differences between pillow heights 2 and 3 in any muscle activity. Comfort evaluation: Height 2 was considered the most comfortable (*P* < 0.001) compared with heights 1 and 3, and height 1 the least comfortable (*P* < 0.001) compared with the other heights. Conclusion: It was found that there is an association among pillow height, myoelectric activity, and comfort. (Height 1: 5 cm; Height 2: 10 cm; Height 3: 14 cm; EMG: electromyographic)
Wang et al. (2015) [14]	Double-blind randomized crossover trial	To study the effect of pillow size preference on the strength and electromyographic (EMG) signals of the upper extremity muscle.	The two most comfortable pillows were associated with significantly larger maximal EDC force than the two worst pillows. However, no significant differences in EMG were observed between pillows. No statistically significant correlation was found between anthropometric parameters and pillow height preference.Anatomical body measurements are not good predictors of optimal pillow height. As EDC muscle strength is affected by pillow height preference, maximal EDC muscle strength may be a useful complement for selecting the optimal pillow size. (EDC: extensor digitorum communis; EMG: electromyographic)
